# Epilepsy and Crime

**Published:** 1903-12-19

**Authors:** 


### The Hospital.

A Journal of

The Medical Sciences and Hospital Administration.

Vol. XXXV.—No. 899. DECEMBER 19, 1903.

### Epilepsy and Crime.

Last week, at the Midland Assizes, a prisoner, who was known to have been an epileptic, was tried for murder and found guilty; the jury, although dismissing the plea of insanity which had been raised, recommended the prisoner to mercy. As to the fact of the murder there was no room for doubt, and the circumstances need not be referred to here; but the case once more brings into prominence the question of responsibility or irresponsibility of an epileptic for crimes committed by him. Here the evidence went to show that the prisoner had been subject to epileptic fits; but there was no record of the occurrence of any for a period of four years or longer. It should be remembered that such attacks might have taken place during the night, and be unknown to everyone except the man himself, and even he might not be always conscious of having had them; or the disease may have assumed the form of *petit mal*. What, if any, medical treatment had the prisoner been subjected to during the few years previous? That question should be answered.

The percentage of epileptics who become certifiably insane is a very large one, and long before the disease reaches this point, the physiological critic can distinguish the difference between, perhaps, a still higher percentage of them and the perfectly normal individal. It is these border-land cases in which the lay mind finds it so difficult to admit the plea of irresponsibility for a crime committed. It has been well observed that the border-land between sanity and insanity is so thin that even experienced persons who have been all their lives associated with the insane may be deceived when in reality the mind is unsound and the patient unsafe, and we may add that in no form of insanity is that border-land so ill-defined as in epilepsy.

The signs of homicidal mania have been enumerated by many authors, and by none more clearly than Esquirol, who states that the main distinctive features between the sane and the insane criminal are that the homicidal maniac has never any accomplice; that he has no motive or a delusive motive only; that his victim is either completely indifferent to him, or is one near and dear to him. The maniac may or may not conceal his crime; perhaps most frequently he does not; but when the above-named distinctions, or the majority of them, are present, it is almost certain that insane irresponsibility existed at the time the crime was committed. Both the sane and the insane murderer may carefully and methodically plan, and carefully and methodically carry out the crime.

In the case which has called up these remarks it was suggested that the prisoner committed the crime in a paroxysm of masked epilepsy; and the statement that the ordinary epileptic seizures were supposed to have been absent for some years, renders this a very probable deduction. Of all cases found in our county asylums, epileptics are the most quarrelsome, the most untruthful, the most prone to committing assaults, the most likely to show evidence of ungovernable temper, and yet shortly before or shortly after these ebullitions it might be extremely difficult to formulate legal proof of insanity. In fact in a considerable number of such cases the homicidal act is the chief evidence of insanity, and sometimes it may be the only tangible evidence.

Many diseases affect the mind temporarily or permanently after the patient is aware that he is suffering from one of these diseases, and very often there is no mental failing until this knowledge has been obtained; but of all diseases which produce this loss of mental balance, epilepsy is one of the most potent. Hence we find that sooner or later a large number of epileptics become mentally unsound; but for a long time this mental unsoundness may not show itself in distinct delusions, or in unmistakably insane acts; and it is only the presence of one or of both of these conditions which conveys proof of insanity to the minds of the jury or it may be to the mind of the judge himself. An action, if it be not in their judgment an insane action, weighs but little with them. An epileptic who intentionally seriously maims himself is, of course, a lunatic, because no one but a lunatic would so act; but let the act be committed on some other person, especially if there have been a previous quarrel, and the act is not accepted as evidence of insanity at all.

If the trial be one for murder it is more than probable that that venerable bore of our youth, that legal shibboleth—could the lunatic distinguish between right and wrong—will be dragged into the question, although it has little more to do with the medical definition of insane responsibility than it has with the diagnosis of cancer or consumption. It is not the mere knowledge of right and wrong that is the crucial point: it is the power to choose between them, and no epileptic should ever be executed until the most searching inquiry into his mental state has been made. And here a difficulty presents itself in nearly all murder cases where the plea of insanity is raised, as it is practically certain that no medical examination has been made by an expert until days, possibly weeks, have elapsed since the commission of the crime. In the meantime the prisoner has been leading a perfectly regular life, with reasonable food and no alcohol, with the result that although undoubted evidence of insanity may have existed at one time, there is none when the specialist sees the prisoner. He may reason correctly from hearsay and from analogy that such evidence did exist at the time of the assault; he may believe that the insane paroxysm would reappear under similar conditions; but the only evidence admissible in Court will be that form of evidence which is more likely to injure the prisoner than to help him. We are of opinion that it should be an invariable rule that all persons charged with murder should be examined by two specialists in mental disease within a few hours of their apprehension, and that such examination should be supplemented by as complete a family history of the prisoner as can be obtained. In a great many cases this early examination would settle the question of the sanity or insanity of the prisoner.

